# Warm versus cold cardioplegia in cardiac surgery: A meta-analysis with trial sequential analysis

**DOI:** 10.1016/j.xjon.2021.03.011

**Published:** 2021-03-31

**Authors:** Thompson Ka Ming Kot, Jeffrey Shi Kai Chan, Saied Froghi, Dawnie Ho Hei Lau, Kara Morgan, Francesco Magni, Amer Harky

**Affiliations:** a Faculty of Medicine, The Chinese University of Hong Kong, Shatin, New Territories, Hong Kong; b Department of Anaesthesia and Intensive Care, Prince of Wales Hospital, Shatin, New Territories, Hong Kong; c Division of Cardiology, Department of Medicine and Therapeutics, Prince of Wales Hospital, Hong Kong; d Division of Surgery and Interventional Sciences, Royal Free Hospital, University College London, London, United Kingdom; e Department of Cardiology, Manchester Royal Infirmary, Manchester, United Kingdom; f Faculty of Biology, Medicine & Health, Division of Pharmacy & Optometry, School of Health Sciences, The University of Manchester, Manchester, United Kingdom; g Faculty of Medicine, University College London, London, United Kingdom; h Department of Cardiothoracic Surgery, Liverpool Heart and Chest Hospital, Liverpool, United Kingdom

**Keywords:** cardiac surgeries, cold cardioplegia, warm cardioplegia, meta-analysis, trial sequential analysis, AF, atrial fibrillation, AKI, acute kidney injury, CABG, coronary artery bypass graft, CI, confidence interval, IABP, intra-aortic balloon pump, ICU, intensive care unit, LCOS, low cardiac output syndrome, LOS, length of stay, MI, myocardial infarction, NOS, Newcastle–Ottawa Quality Assessment Scale, PRISMA, Preferred Reporting Items for Systematic Reviews and Meta-Analyses, RCT, randomized controlled trial, RR, risk ratio, TSA, trial sequential analysis, WMD, weighted mean difference

## Abstract

**Objective:**

This meta-analysis aimed to compare clinical outcomes of warm and cold cardioplegia in cardiac surgeries in adult patients, with trial sequential analysis (TSA) used to determine the conclusiveness of the results.

**Methods:**

Electronic searches were performed on PubMed, Medline, Scopus, EMBASE, and Cochrane library to identify all studies that compared warm and cold cardioplegia in cardiac surgeries. Primary end points were in-hospital or 30-day mortality, myocardial infarction, low cardiac output syndrome, intra-aortic balloon pump use, stroke, and new atrial fibrillation. Secondary end points were acute kidney injury, hospital length of stay, and intensive care unit length of stay. Prespecified subgroup analyses were performed for (1) studies published since publication of Fan and colleagues in 2010, (2) randomized controlled studies, (3) studies with low risk of bias, (4) coronary artery bypass graft surgeries, and (5) studies with cold blood versus those with cold crystalloid cardioplegia. TSA was performed to determine conclusiveness of the results, using on all outcomes without significant heterogeneity from studies of low risk of bias.

**Results:**

No significant differences were found between post-operative rates of mortality, myocardial infarction, low cardiac output syndrome, intra-aortic balloon pump use, stroke, new atrial fibrillation, and acute kidney injury between warm and cold cardioplegia. TSA concluded that current evidence was sufficient to rule out a 20% relative risk reduction in these outcomes.

**Conclusions:**

Concerning safety outcomes, current evidence suggests that the choice between warm and cold cardioplegia remains in the surgeon's preference.

## Graphical abstract

Warm versus cold cardioplegia in cardiac surgery: A meta-analysis with trial sequential analysis. Forty-six studies with 15,428 patients were included in the analysis (35 randomized controlled trials + 11 observational studies). No significant differences were found between 2 arms in postoperative mortality, myocardial infarction, low cardiac output syndrome, intra-aortic balloon pump use, stroke, and new atrial fibrillation, as shown in the forest plots. Trial sequential analysis of mortality was shown signifying that current evidences were conclusive. In conclusion, choice of warm versus cold cardioplegia remains the surgeon's preference.

**Figure:**
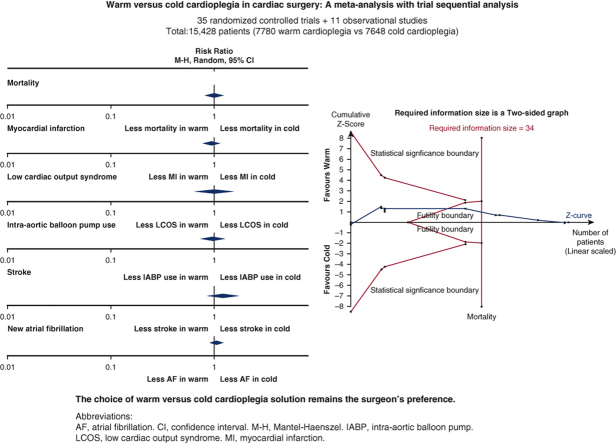

**Figure:**
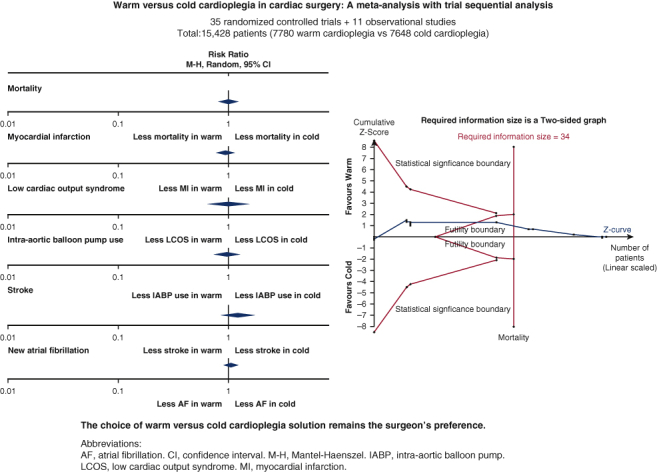
No significant differences were found in major postoperative outcomes between warm and cold cardioplegia. *M-H*, Mantel-Haenszel; *CI*, confidence interval.

**Central Message**

The choice between warm and cold cardioplegia remains the surgeon's preference.

**Perspective**

This systematic review and meta-analysis showed no differences between postoperative rates of mortality, MI, LCOS, IABP use, stroke, new AF, and AKI between warm and cold cardioplegia. TSA concluded that current evidence was sufficient to rule out a 20% relative risk reduction in these outcomes.

See Commentary on page 191.

Cardioplegia allows for a still operative field, which is important in cardiac surgeries. There are various forms of cardioplegic solutions nowadays, which can be administrated in different ways. These include blood versus crystalloid, cold versus warm, intermittent versus continuous, antegrade versus retrograde versus combined, and terminal warm shot cardioplegia.

Concerns have long been raised about the clinical outcomes of different forms of cardioplegia. Since the 1970s, there has been debate over the optimal temperature for cardioplegic solutions. Cold cardioplegia has been used to maximize myocardial cooling and metabolic inhibition. In contrast, warm cardioplegia was proposed as an alternative to meet the energy demands of the arrested heart; lower the risk of membrane destabilization, intracellular edema, calcium sequestration, and time for heart rewarming; and decrease the risk of reperfusion injury. Besides, blood was considered to be better than crystalloid cardioplegia due to its greater oxygen-carrying and buffering capacity, better microvascular flow secondary to rheologic effects, and less associated intracellular edema.^1^

The Warm Heart Investigators^2^ conducted a randomized controlled trial (RCT) of 1732 patients in 1994. They demonstrated a significant reduction in postoperative low cardiac output syndrome (LCOS) in the warm cardioplegia group, without significant differences in 30-day all-cause mortality, postoperative myocardial infarction (MI), and stroke. A meta-analysis on RCTs by Fan and colleagues^3^ showed no significant difference in the clinical outcomes investigated. However, it was unclear whether the results were conclusive. This systemic review and meta-analysis aimed to compare clinical outcomes of warm versus cold cardioplegia in adult cardiac surgeries, updating the meta-analysis by Fan and colleagues^3^ with more recent evidence, further analyzing the conclusiveness of the results.

## Methods

This systematic review and meta-analysis was conducted according to the Preferred Reporting Items for Systematic Reviews and Meta-Analyses (PRISMA) statement and methods stipulated in the Cochrane Handbook for Systematic Review of Interventions.^4^^,^^5^ It has been submitted to PROSPERO with a registration number of CRD42020171613 but had not been approved as of the time of submission.

### Search Strategy and Selection Criteria

Electronic searches were performed on PubMed, Medline, Scopus, EMBASE, and Cochrane library to identify all studies comparing warm and cold cardioplegia in cardiac surgeries regardless of publication type or language. All databases were searched since the search of previous meta-analysis (Fan and colleagues^3^) on the topic, up until June 27, 2020. A search was also conducted on ClinicalTrials.gov to identify ongoing or unpublished clinical trials. The search string used was ([warm OR normothermia OR normothermic OR cold OR hypothermia OR hypothermic] AND [cardioplegia OR "myocardial protection"] AND [valve OR valvular OR AVR OR MVR OR DVR OR TVR OR PVR OR "coronary artery bypass graft" OR "coronary artery bypass grafting" OR CABG OR "vein graft" OR "bypass graft" OR "surgical revascularization"]). All search terms searched as both key words and Medical Subject Headings terms to maximize sensitivity. Reference lists of papers found in the literature search were manually searched to assess suitability for inclusion in this review.

Three reviewers performed literature screening (T.K.M.K., J.S.K.C., Shaik Ashraf Bin Shaik Ismail). Articles were first screened based on their titles and abstracts. Full texts of all identified articles were then retrieved and systemically assessed using the inclusion and exclusion criteria for further study. Conflicts over inclusion were resolved by consensus. Articles were deemed eligible for inclusion if warm cardioplegia was compared against cold cardioplegia in cardiac surgeries. Noncomparative studies, conference abstracts or papers, articles involving fewer than 5 patients, and studies including patients younger than 18 years of age were excluded. Studies not reporting any of the end points specified herein were also excluded. Warm cardioplegia was defined as 28°C to 37°C, whereas cold cardioplegia was defined as 4°C to 15°C.

Primary end points were in-hospital or 30-day mortality, MI, LCOS, intra-aortic balloon pump (IABP) use, stroke, and new atrial fibrillation (AF). Secondary end points were acute kidney injury (AKI), hospital length of stay (LOS), and intensive care unit (ICU) LOS. Summary estimates were extracted manually from included studies. Only the most updated data were included wherever duplicate data existed. Study authors were contacted where necessary. Data reported by previous meta-analysis by Fan and colleagues in 2010^3^ were also extracted from published Forest plots. Conflicts over data extraction were resolved by consensus.

### Statistical Analysis

All included studies were critically appraised by the modified Jadad scale for RCTs or the Newcastle–Ottawa Quality Assessment Scale (NOS) for observational studies. The modified Jadad scale is a numeral scale with components addressing randomization, blinding, selection, adverse effects assessment, and statistical methods. It is described in detail in Table E1. The NOS assessed cohort studies according to selection, comparability, and outcome and is detailed in Table E2. The meta-analysis by Fan and colleagues in 2010^3^ was critically appraised by the AMSTAR 2, which is a critical appraisal tool for systematic reviews that includes randomized or nonrandomized studies of health care interventions.^6^

All statistical analyses were a priori, specified before the start of data extraction. Odds ratios and 95% confidence intervals (CIs) or weighted mean differences (WMDs) and 95% CIs were used as the main summary measures for baseline characteristics, whereas relative risks (RRs) and 95% CIs or WMD and 95% CIs were used as main summary measures for the outcomes studied. Discrete variables were pooled using the Mantel–Haenszel method with RR as the effect measure. Continuous variables were pooled using the inverse variance method with WMD as the effect measure. Sensitivity analysis is performed by the leave-one-out method. Prespecified subgroup analysis was performed on (1) studies published since publication of Fan and colleagues in 2010^3^; (2) RCTs; (3) studies with low risk of bias, defined by 5 or 7 score or more in modified Jadad scale or NOS, respectively; (4) coronary artery bypass graft (CABG) surgeries; and (5) studies with cold blood versus those with cold crystalloid cardioplegia.

Heterogeneity was assessed by the Cochran's Q test and I^2^ statistics. All variables were analyzed using the DerSimonian–Laird random effects model. For variables reported by at least 10 studies, publication bias was assessed visually by funnel plot.

Trial sequential analysis (TSA) can be used to assess conclusiveness of meta-analytical findings. As evidence accumulates, random errors also accumulates and they may incidentally lead to “significant” results reported in meta-analysis. Meta-analyses of cardiovascular and anesthesiologic interventions have many false positions and negative results due to the low statistical power of the meta-analysis when the required number of participants or trials has not been reached, which can be addressed by TSA.^7^ Trials were included in chronological order and handled as interim analysis relative to the required information size, which is defined as the number of participants and events necessary to detect or reject an a priori assumed intervention effect in meta-analysis. Statistical techniques were used to adjust the CI of point estimate and to increase the threshold for statistical significance based on effect to be observed, incidence of outcome in control arm, information size, and heterogeneity.^8^ It was performed on all outcomes without significant heterogeneity, from studies of low risk of bias. Z-score curve was generated by plotting cumulative Z scores with new study data. A Z-score curve crossing either of statistical significance boundaries (ie, the pair of outer oblique lines) implies that the statistically significant data is conclusive, whereas crossing either of the futility boundaries (ie, inner oblique lines) implies that the statistically insignificant data is conclusive. If the curve crosses the required information size boundary (ie, the vertical line), all observations are said to be conclusive.^8^ All available statistical information (Fisher information) was used. The Z-score threshold was adjusted using the O'Brien–Fleming alpha-spending function. Studies reporting no events were handled by adding a constant (1) to both arms. Required information sizes were estimated from an RR reduction of 20%, chosen to represent a clinically meaningful effect. Incidences were calculated from all studies reporting the outcome of interest. Heterogeneity and variance adjustments were estimated from all included studies in TSA. A prespecified permissible 2-sided type 1 error (α) of 5% and type 2 error (β) of 20% were used, therefore giving a power of 80%.

All *P* values are 2-sided. The meta-analytical component was performed using Review Manager (RevMan), version 5.3 (Copenhagen: The Nordic Cochrane Centre, The Cochrane Collaboration, 2014). The TSA component was performed using the Copenhagen trial unit, TSA software, version 0.9.5.10 Beta.

## Results

**Figure 1:**
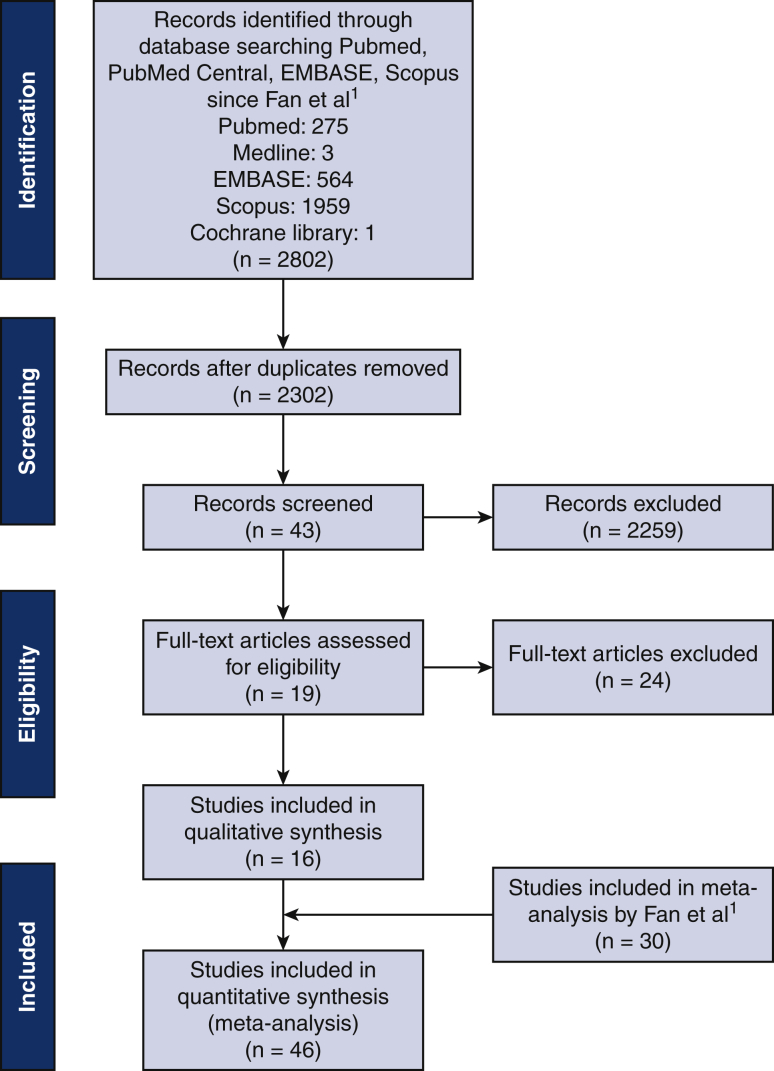
The Preferred Reporting Items for Systematic Reviews and Meta-Analyses (PRISMA) flow diagram.

The literature search is summarized in a PRISMA diagram (Figure 1). A total of 2802 nonduplicate citations were identified; after full-text screening of 43 papers, only 16 papers published after 2009 met the inclusion criteria. Together with the papers included by Fan and colleagues,^3^ there were 35 RCTs and 11 observational studies (Table 1). A total of 15,428 patients were included (7780 in warm cardioplegia arm, 7648 in cold cardioplegia arm). Electronic search of ClinicalTrials.gov revealed an ongoing RCT (NCT04203680) comparing cold histidine–tryptophan–ketoglutarate solution versus warm blood cardioplegia in CABG, with 30-day mortality as the primary outcome.

**Table 1:** Characteristics of studies included

Author	Year	Surgery type	No. of patients (warm cohort)	No. of patients (cold cohort)	Warm cardioplegia temperature, °C	Cold cardioplegia temperature, °C	Key finding	Risk of bias -MJS (/8) NOS (/9)
Ali et al^E2^	1994	CABG, valve	38	CB: 38	37	10	Intermittent warm blood was as safe as cold blood cardioplegia when the aortic crossclamp time was less than 90 min.	3/8
Ascione et al^E3^	2002	Valve	19	CB: 16	34	6-8	Warm blood cardioplegia was associated with more ischemic stress and myocardial injury, as compared with cold blood cardioplegia in patients with aortic stenosis undergoing valvular replacement.	5/8
Raza Baig et al^E37^	2015	CABG	94	CB: 121	NR	NR	Intermittent antegrade warm blood cardioplegia was associated with better myocardial protection in early postoperative period.	9/9
Baron et al^E4^	2003	CABG	48	CB: 21	37	15	Warm and cold blood cardioplegia were comparable in terms of postoperative complications and mortality rate.	3/8
Candilio et al^E38^	2014	CABG	10	CB: 28	NR	NR	Antegrade retrograde cardioplegia was associated with less perioperative myocardial infarction compared with antegrade cardioplegia.	9/9
Chello et al^E5^	1997	CABG	20	CB: 20	37	5	Warm cardioplegia was associated with increased activation of complement and neutrophils compared with cold cardioplegia.	2/8
Chello et al^E6^	2003	CABG	20	CB: 20	37	5	Intermittent warm cardioplegia was associated with better myocardial protection, and increased HSP72 expression.	4/8
Chocron et al^E7^	2000	CABG	45	CB: 45	37	8	Intermittent warm blood cardioplegia was associated with comparable postoperative complications and fewer myocardial injuries in low-risk patients.	6/8
Curtis et al^E8^	1996	CABG	40	CB: 38	NR	4	Warm cardioplegia was associated with comparable morbidity and mortality compared with cold cardioplegia.	4/8
Dar et al^E9^	2005	CABG	20	CC: 10	37	4	Antegrade with retrograde warm blood cardioplegia was associated with lower postoperative cardiac enzymes compared with antegrade cardioplegia.	4/8
De Jonge et al^E39^	2015	CABG	2585	CC: 2585	37	4	Blood cardioplegia was an independent risk factor for increased creatine kinase-MB after CABG.	8/9
Elwatidy et al^E10^	1999	CABG	47	CB: 40 CC: 41	28-30	CB: 8 CC: 4	Warm blood cardioplegia was associated with better metabolic and functional recovery, without significant differences in morbidity and mortality.	4/8
Engelman et al^E11^	1996	CABG	93	CB: 37	32/37	8-10	Warm cardioplegia was associated with more activation of fibrinolytic potential and fewer neurologic adverse events.	8/8
Franke et al^E12^	2003	CABG	100	CB: 100	33	4	Intermittent antegrade warm blood cardioplegia was associated with lower postoperative cardiac enzymes.	6/8
Gaudino et al^E13^	2013	Valve	29	CC: 31	37	0	Warm cardioplegia was associated with better right ventricular protection compared with one-shot histidine–tryptophane–ketoglutarate cardioplegia solution.	7/8
Hayashida et al^E14^	1994	CABG	48	CB: 24	W: 37 L: 29	8	Warm cardioplegia was associated with more lactate and acid washout with reperfusion and better cardiac function postoperatively.	4/8
Hayashida et al^E15^	1995	CABG	28	CB: 14	W: 37 L: 29	9	Warm and tepid cardioplegia were associated with better cardiac function postoperatively.	4/8
Honkonen et al^E16^	1997	CABG	15	CB: 14	37	5-7	Warm cardioplegia was associated with better recovery of right ventricular function in terms of ejection fraction and preload related stroke work and less postoperative cardiac enzymes release.	4/8
Isomura et al^E17^	1995	CABG	29	CC: 26	26-37	4	Warm cardioplegia was associated with comparable myocardial protection and clinical outcomes compared with cold cardioplegia.	3/8
Jacquet et al^E18^	1999	CABG	108	CC: 92	37	NR	Intermittent antegrade warm blood cardioplegia was associated with lower postoperative cardiac enzyme release.	5/8
Kammerer et al^E19^	2010	Valve	52	CC: 55	35	4	Warm blood cardioplegia was associated with significantly greater mortality rate compared with cold crystalloid cardioplegia.	4/8
Kuhn et al^E20^	2015	CABG	36	CB: 32	37	4-6	Intermittent warm cardioplegia was associated with greater extent of endothelial injury and comparable rates of clinical end points compared with cold cardioplegia.	7/8
Kuhn et al^E40^	2018	CABG	212	CB: 212	37	4-6	No significant differences were found in myocardial protection and similar postoperative adverse events between Buckberg and Calafiore cardioplegia.	8/9
Lajos et al^E21^	1993	CABG	54	CB: 54 CB: 55	37	NR	Intermittent cold cardioplegia provided a clearer operative field compared with continuous warm cardioplegia, without better myocardial protection.	3/8
Landymore et al^E22^	1996	CABG	20	CB: 20	37	8	Warm cardioplegia was associated with comparable myocardial metabolic and functional recovery and postoperative adverse events compared with cold cardioplegia.	5/8
Maccherini et al^E23^	1995	CABG	50	CB: 50	37	4-8	Warm blood cardioplegia was associated with less pleural effusions and thoracentesis related to hypothermia.	2/8
Martin et al^E24^	1994	CABG	493	CC: 508	≥35	≤8	Warm cardioplegia was associated with more neurologic events, as defined as stroke and encephalopathy, compared with cold cardioplegia.	4/8
Mourad et al^E41^	2016	CABG	50	CC: 50	NR	NR	Antegrade warm blood cardioplegia was associated with lower postoperative cardiac enzymes release.	9/9
Nardi et al^E42^	2018	CABG Valve	159	CC: 32	35-36	4	Cold crystalloid cardioplegia was associated with less postoperative cardiac enzymes release and comparable postoperative clinical outcomes compared with warm blood cardioplegia.	8/9
Nardi et al^E43^	2018	CABG	297	CC: 33	34-35	4	No significant differences were found in postoperative clinical outcomes between warm and cold cardioplegia in patients undergoing CABG.	8/9
Pelletier et al^E25^	1994	CABG	100	CB: 100	NR	NR	Warm cardioplegia was associated with less postoperative cardiac enzymes release, and comparable rates of mortality and myocardial infarction compared with cold cardioplegia.	6.5/8
Pepper et al^E26^	1995	Valve	15	CB: 17 CC: 15	37	4	Blood cardioplegia was associated with greater thiol level.	3.5/8
Plicner et al^E44^	2017	CABG	124	CC: 114	37	4	No significant differences were found in postoperative systemic inflammatory response and oxidative stress, between warm and cold cardioplegia.	9/9
Rashid et al^E27^	1994	CABG	137	CB: 144	37	4-6	No significant differences were found between warm and cold cardioplegia for myocardial protection and postoperative adverse clinical outcomes.	2/8
Rashid et al^E28^	1995	CABG	58	CB: 50	37	8	Warm cardioplegia was associated with comparable myocardial protection in patients with left ventricular dysfunction in CABG compared with cold cardioplegia.	2/8
Rosu et al^E45^	2012	CABG	54	CB: 84	27.6	10.1	Tepid cardioplegia was associated with a greater rate of LCOS compared with cold cardioplegia.	8/9
Saclı et al^E29^	2019	CABG	20	CB: 28	28.4	13.7	Cold cardioplegia was associated with less myocardial injury and postoperative morbidity compared with warm cardioplegia.	3/8
Şirlak et al^E30^	2003	CABG	50	CC: 50	32-34	4-6	No significant differences were found in postoperative cardiac enzymes release between tepid and cold cardioplegia.	5/8
Sirvinskas et al^E31^	2005	CABG	101	CC: 55	W: 37 L: 28-30	4	Intermittent antegrade warm cardioplegia was associated with lower postoperative troponin T release, shorter duration of tracheal intubation, and hospital stay.	6/8
The Warm Heart Investigator^E32^	1994	CABG	860	CB: 872	37	5-8	Warm cardioplegia was associated with significantly lower rates of LCOS and comparable rates of mortality, stroke, and myocardial infarction compared with cold cardioplegia.	6.5/8
Trescher et al^E46^	2017	CABG Valve	610	CB: 1578	32-34	6-8	No significant differences were found in myocardial protection between intermittent warm and cold blood cardioplegia.	8/9
Ucak et al^E33^	2019	CABG	185	CC: 112	33-34	4	No significant differences were found in clinical outcomes between intermittent warm and cold cardioplegia.	5/8
Yau et al^E34^	1992	CABG	48	CB: 26	37	5	No significant differences were found in clinical outcomes between warm and cold cardioplegia.	3/8
Yau et al^E35^	1993	CABG	43	CB: 64	37	5	Warm cardioplegia was associated with comparable morbidity and mortality compared with cold cardioplegia.	5/8
Yang et al^E36^	1994	Valve	10	CC: 10	37	4	No significant differences were found in clinical outcomes between warm and cold cardioplegia.	2/8
Zeriouh et al^E47^	2015	CABG	506	CB: 176	37	4-6	Intermittent warm cardioplegia was associated with comparable long-term outcomes as compared with intermittent cold cardioplegia.	9/9

*MJS*, Modified Jadad scale; *NOS*, Newcastle–Ottawa Quality Assessment Scale; *CABG*, coronary artery bypass graft; *CB*, cold blood; *NR*, not reported; *CC*, cold crystalloid; *W*, warm; *L*, lukewarm; *LCOS*, low cardiac output syndrome.

Critical appraisal of the included studies was performed using the modified Jadad scale or NOS, as summarized in Table E1 and Table E2, respectively. Overall, 18 of 35 RCTs scored 5 points or greater in modified Jadad scale, with all observational studies scoring 7 points or greater in NOS, and were classified as low risk of bias. Significant proportion of studies included before 2009 were classified as having high risk of bias, mainly due to inappropriate randomization methods and nonblinded studies.

Critical appraisal of meta-analysis by Fan and colleagues in 2010^3^ was performed using the AMSTAR 2 tool,^6^ as summarized in Online data supplement. It showed that the systemic review was of moderate quality.

Baseline characteristics of included patients in studies after previous meta-analysis were summarized in Table E3. Other related baseline characteristics (smoker, European System for Cardiac Operative Risk Evaluation, European System for Cardiac Operative Risk Evaluation II, dyslipidemia, peripheral vascular disease, chronic kidney disease, previous AF, chronic obstructive pulmonary disease, previous stroke, previous MI) were not reported, as they were included by fewer than 10 studies.

A pairing table (Table E4) was constructed to indicate outcomes reported by individual studies. All primary outcomes were supported by at least 15 studies (mortality 31, MI 32, LCOS 15, IABP use 20, stroke 17, new AF 17), whereas secondary outcomes were supported at least 7 studies (AKI 7, hospital LOS 9, ICU LOS 10).

**Figure 2:**
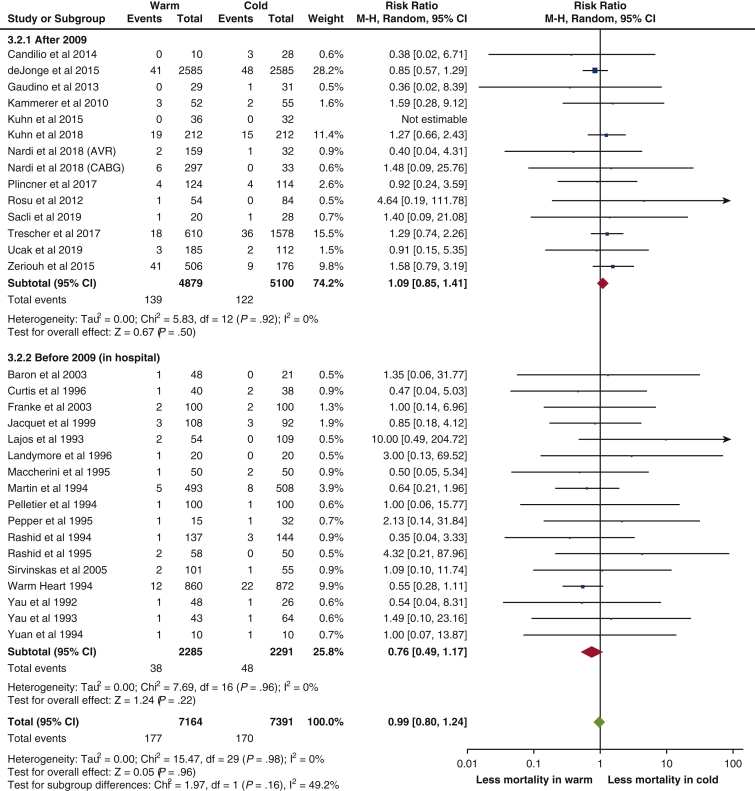
Forest plot for mortality. *M-H*, Mantel–Haenszel; *CI*, confidence interval.

**Figure 3:**
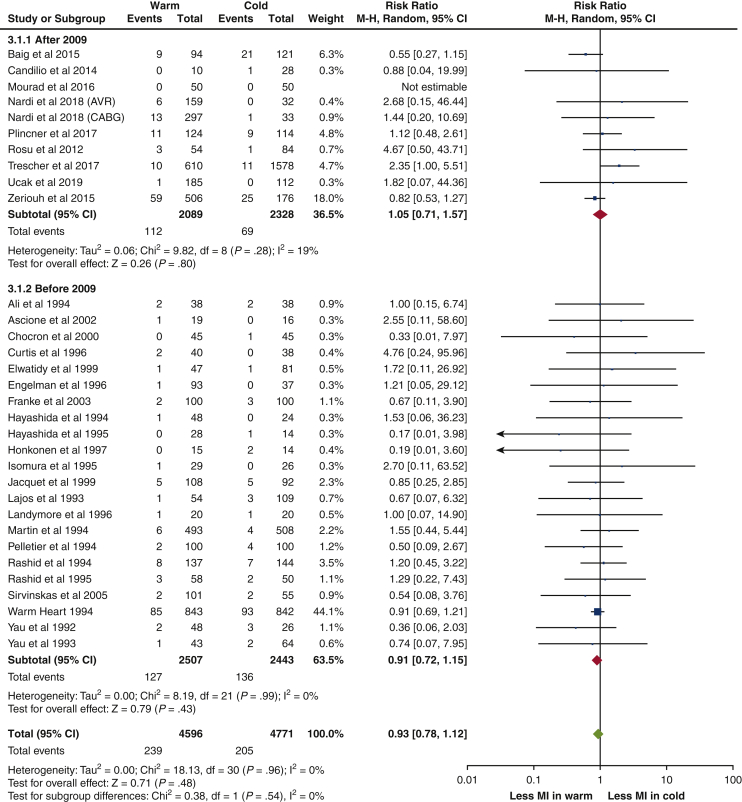
Forest plot for MI. *M-H*, Mantel–Haenszel; *CI*, confidence interval; *MI*, myocardial infarction.

There were no statistically significant differences in all outcomes (mortality, MI, LCOS, IABP use, stroke, new AF, AKI, hospital LOS, and ICU LOS) between warm and cold cardioplegia, with results summarized in Table 2.Forest plots of outcomes reported by most studies (ie, mortality and MI) were shown in Figures 2 and 3, respectively. None of the primary outcomes exhibited significant heterogeneity. Only hospital LOS and ICU LOS had significant heterogeneity.

**Table 2:** Summary of primary and secondary outcomes

	RR or WMD [95% CI]	*P* value	Heterogeneity
Mortality	RR 0.99 [0.80-1.24]	.96	I^2^ = 0%, χ^2^ = 15.47, *P* = .98
MI	RR 0.93 [0.78-1.12]	.48	I^2^ = 0%, χ^2^ = 18.13, *P* = .96
LCOS	RR 0.98 [0.64-1.50]	.92	I^2^ = 36%, χ^2^ = 21.91, *P* = .08
IABP use	RR 0.95 [0.70-1.28]	.72	I^2^ = 0%, χ^2^ = 12.70, *P* = .69
Stroke	RR 1.19 [0.83-1.69]	.35	I^2^ = 0%, χ^2^ = 10.00, *P* = .76
New AF	RR 1.08 [0.92-1.26]	.34	I^2^ = 19%, χ^2^ = 19.79, *P* = .23
AKI	RR 0.94 [0.59-1.48]	.78	I^2^ = 0%, χ^2^ = 5.50, *P* = .48
Hospital LOS	WMD –0.60 [–1.40, 0.20]	.14	I^2^ = 69%, χ^2^ = 22.69, *P* = .002
ICU LOS	WMD –0.12 [–0.56, 0.32]	.60	I^2^ = 88%, χ^2^ = 76.45, *P* < .00001

*RR*, Relative risk; *WMD*, weighted mean difference; *CI*, confidence interval; *MI*, myocardial infarction; *LCOS*, low cardiac output syndrome; *IABP*, intra-aortic balloon pump; *AF*, atrial fibrillation; *AKI*, acute kidney injury; *LOS*, length of stay; *ICU*, intensive care unit.

**Figure E1:**
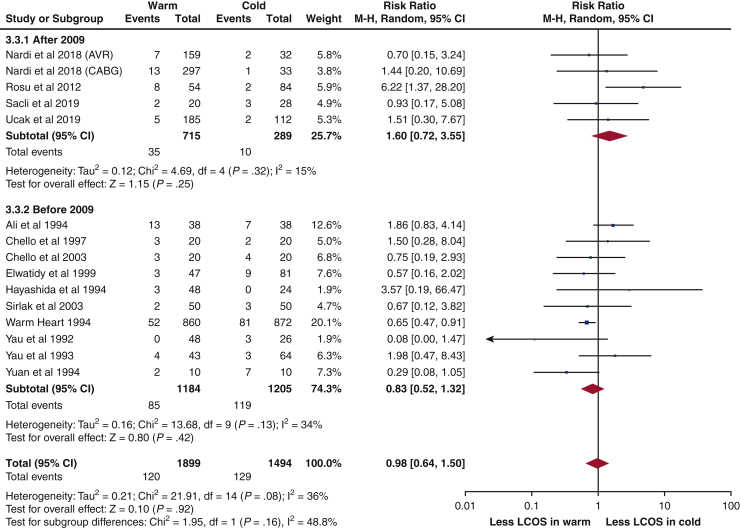
Forest plot for low cardiac output syndrome. Subgroup analysis of studies published after Fan and colleagues.^E1^*M-H*, Mantel–Haenszel; *CI*, confidence interval; *AVR*, aortic valve replacement; *CABG*, coronary artery bypass graft; *LCOS*, low cardiac output syndrome.

**Figure E2:**
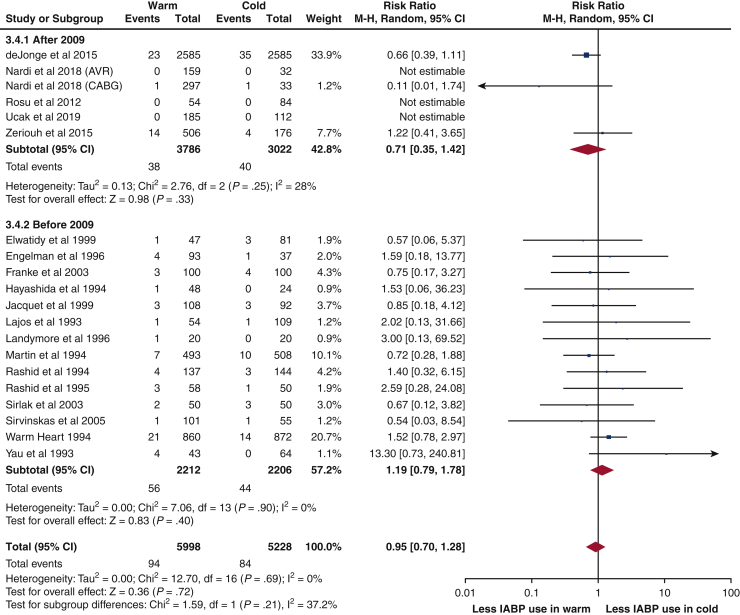
Forest plot for intra-aortic balloon pump use. Subgroup analysis of studies published after Fan and colleagues.^E1^*M-H*, Mantel–Haenszel; *CI*, confidence interval; *AVR*, aortic valve replacement; *CABG*, coronary artery bypass graft; *IABP*, intra-aortic balloon pump.

**Figure E3:**
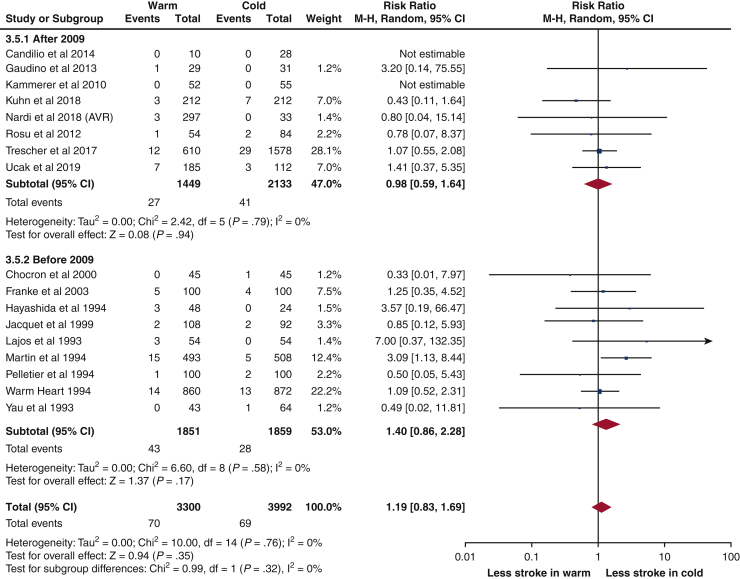
Forest plot for stroke. Subgroup analysis of studies published after Fan and colleagues.^E1^*M-H*, Mantel–Haenszel; *CI*, confidence interval.

**Figure E4:**
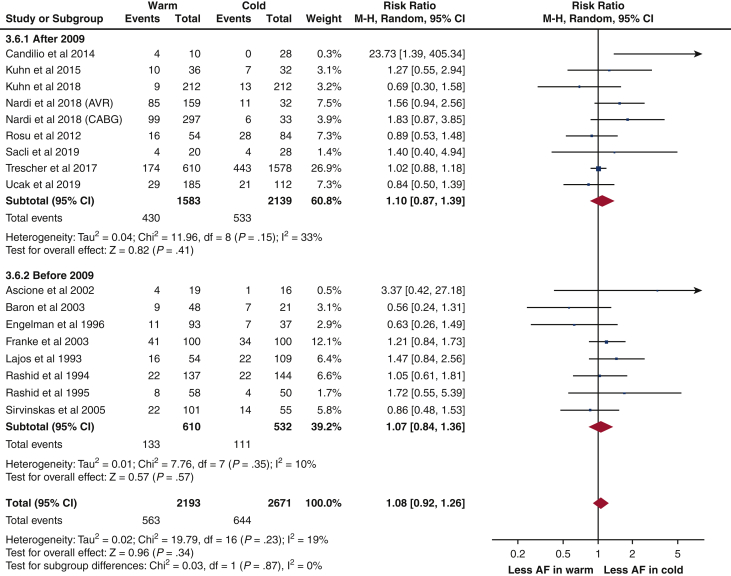
Forest plot for new atrial fibrillation. Subgroup analysis of studies published after Fan and colleagues.^E1^*M-H*, Mantel–Haenszel; *CI*, confidence interval; *AVR*, aortic valve replacement; *CABG*, coronary artery bypass graft; *AF*, atrial fibrillation.

A prespecified subgroup analysis was performed on primary outcomes for studies published since Fan and colleagues in 2010,^3^ with results summarized in Table E5 and forest plots included in Figures 2 and 3 and Figure E1, Figure E2, Figure E3, Figure E4. All of the primary outcomes remained statistically insignificant without significant heterogeneity.

Subgroup analysis were also performed on (1) randomized controlled studies, (2) studies of low risk of bias, (3) CABG surgeries, and (4) studies with cold blood versus those with cold crystalloid cardioplegia, with results summarized in Table 3, Table E6, Table E7, and Table E8, respectively. Most outcomes remained statistically insignificant, with heterogeneity qualitatively unchanged. Exceptions included hospital LOS (WMD –0.84 [–1.59, –0.10], *P* = .03) in studies of low risk of bias; and IABP use (RR 0.65 [0.43-0.99], *P* = .04) in warm blood versus cold crystalloid cardioplegia, both favoring warm cardioplegia.

**Table 3:** Summary of primary and secondary outcomes in randomised controlled studies

	RR or WMD [95% CI]	*P* value	Heterogeneity
Mortality	RR 0.80 [0.54-1.19]	.27	I^2^ = 0%, χ^2^ = 8.76, *P* = .99
MI	RR 0.91 [0.73-1.15]	.45	I^2^ = 0%, χ^2^ = 8.37, *P* = 1.00
LCOS	RR 0.85 [0.57-1.27]	.44	I^2^ = 24%, χ^2^ = 14.43, *P* = .21
IABP use	RR 1.19 [0.82-1.74]	.37	I^2^ = 0%, χ^2^ = 7.06, *P* = .93
Stroke	RR 1.43 [0.91-2.24]	.12	I^2^ = 0%, χ^2^ = 6.85, *P* = .74
New AF	RR 1.06 [0.87-1.28]	.56	I^2^ = 0%, χ^2^ = 8.99, *P* = .53
AKI	RR 0.85 [0.20-3.54]	.82	I^2^ = 0%, χ^2^ = 0.28, *P* = .60
Hospital LOS	WMD −0.44 [−1.54, 0.67]	.44	I^2^ = 47%, χ^2^ = 3.80, *P* = .15
ICU LOS	WMD 0.24 [−0.34, 0.83]	.42	I^2^ = 79%, χ^2^ = 13.99, *P* = .003

*RR*, Relative risk; *WMD*, weighted mean difference; *CI*, confidence interval; *MI*, myocardial infarction; *LCOS*, low cardiac output syndrome; *IABP*, intra-aortic balloon pump; *AF*, atrial fibrillation; *AKI*, acute kidney injury; *LOS*, length of stay; *ICU*, intensive care unit.

**Figure E5:**
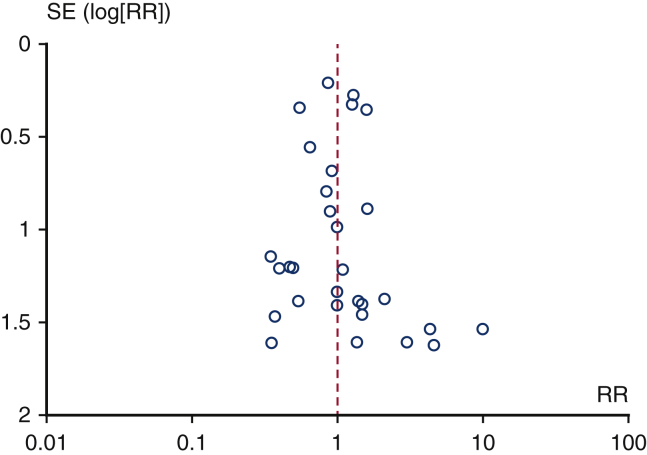
Funnel plot for mortality. *SE*, Standard error; *RR*, relative risk.

**Figure E6:**
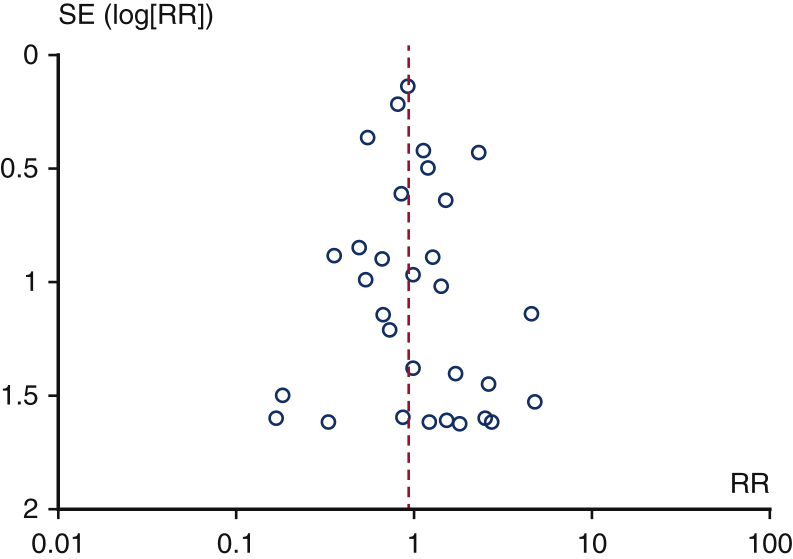
Funnel plot for myocardial infarction. *SE*, Standard error; *RR*, relative risk.

**Figure E7:**
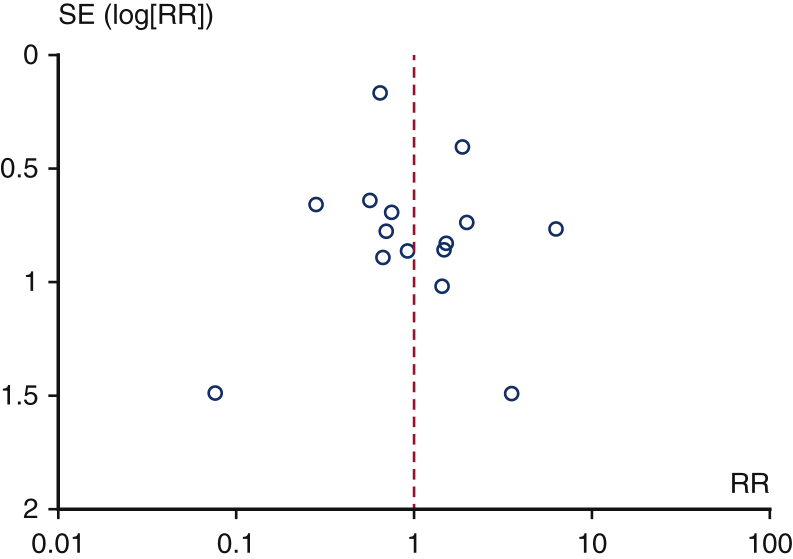
Funnel plot for low cardiac output syndrome. *SE*, Standard error; *RR*, relative risk.

**Figure E8:**
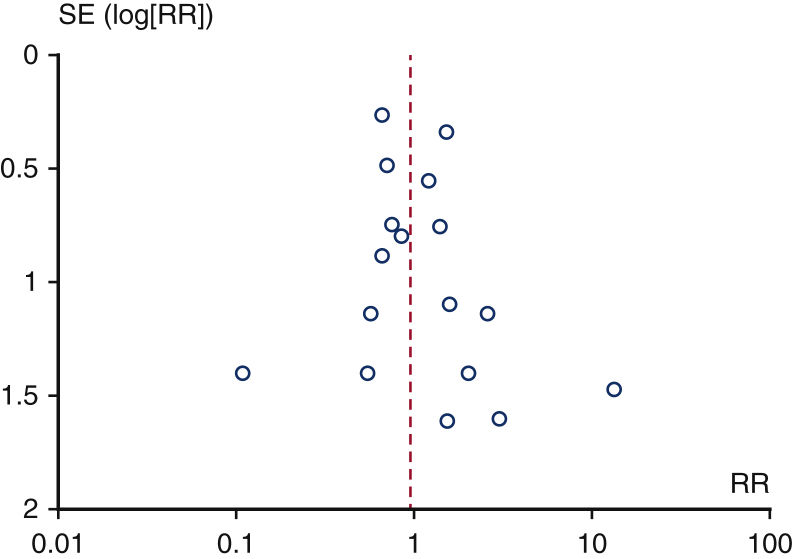
Funnel plot for intra-aortic balloon pump use. *SE*, Standard error; *RR*, relative risk.

**Figure E9:**
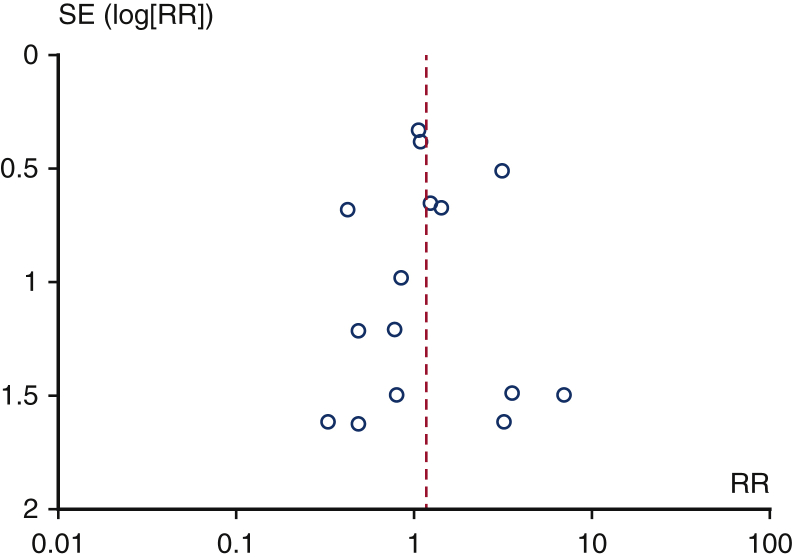
Funnel plot for stroke. *SE*, Standard error; *RR*, relative risk.

**Figure E10:**
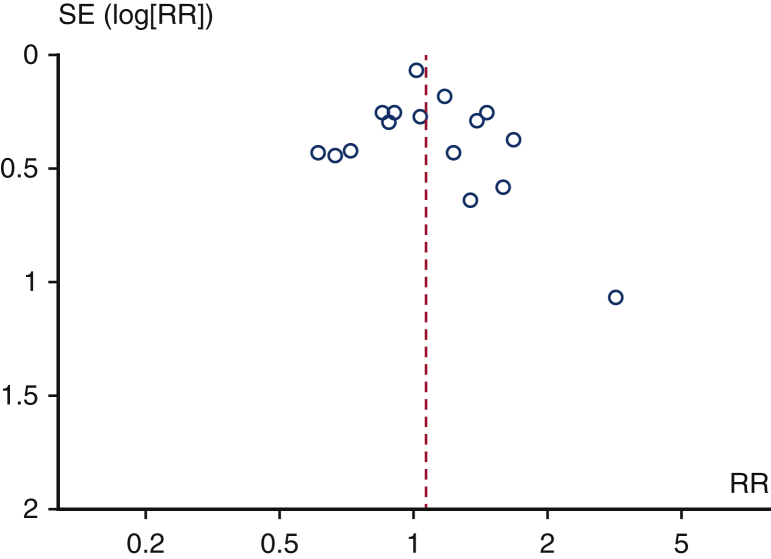
Funnel plot for new atrial fibrillation. *SE*, Standard error; *RR*, relative risk.

**Figure E11:**
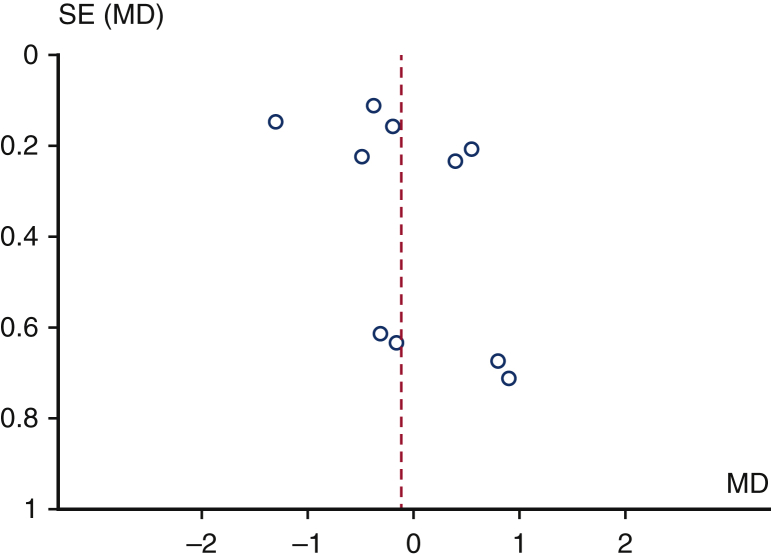
Funnel plot for intensive care unit length of stay. *SE*, Standard error; *MD*, mean difference.

Publication bias was assessed visually by funnel plots for outcomes with at least 10 studies (mortality, MI, LCOS, IABP use, stroke, new AF, and ICU LOS) (Figure E5, Figure E6, Figure E7, Figure E8, Figure E9, Figure E10, Figure E11). No asymmetries were detected, indicating low risk of publication bias.

Sensitivity analysis was performed for all the outcomes using the leave-one-out method. Removal of individual studies from the analysis did not alter the statistical significance, except for the exclusion of Nardi and colleagues^9^ in hospital LOS, which would result in statistically significant (*P* = .04) shorter LOS in warm cardioplegia arm.

**Figure 4:**
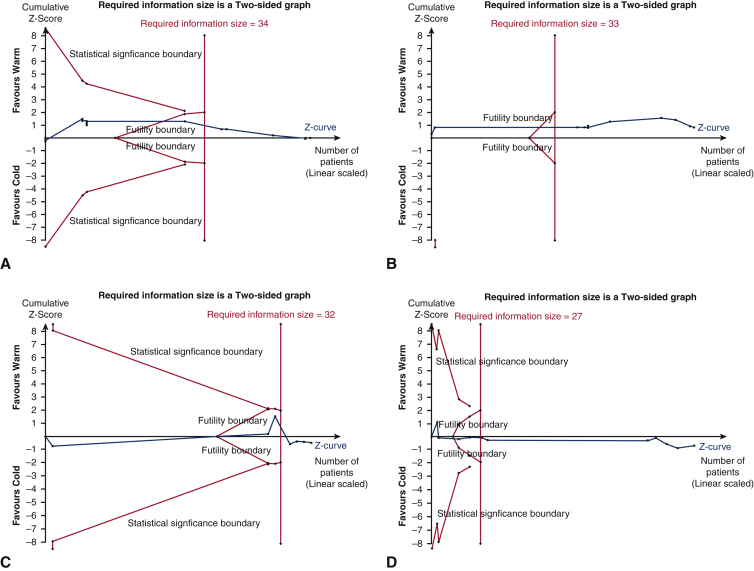
Trial sequential analysis of (A) mortality, (B) myocardial infarction, (C) low cardiac output syndrome, and (D) atrial fibrillation. Z value is the test statistic and |*Z*| = 1.96 corresponds to a *P* = .05. The required information size to detect or reject the 20% relative risk reduction found in random-effects model meta-analysis is calculated using diversity found in meta-analysis, with double-sided α = 0.05 and β = 0.20 (power of 80%).

**Figure 5:**
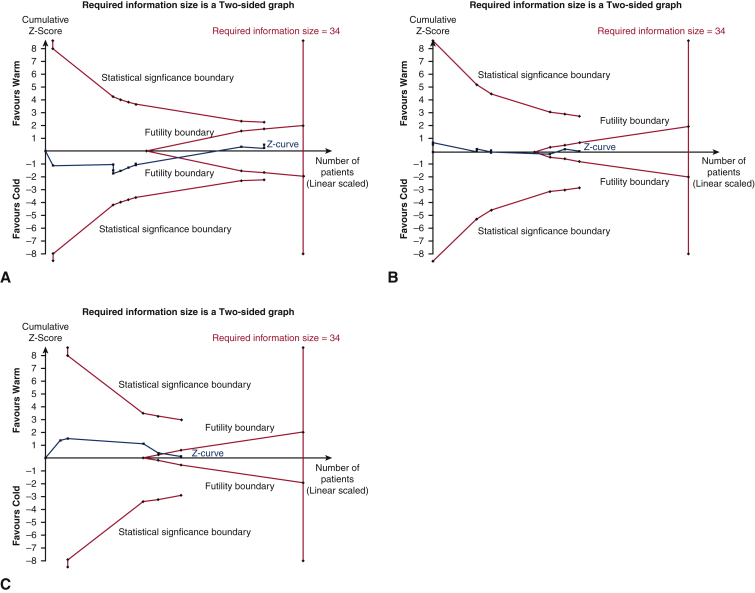
Trial sequential analysis of (A) intra-aortic balloon pump use, (B) stroke, and (C) acute kidney injury. Z value is the test statistic and |*Z*| = 1.96 corresponds to a *P* = .05. The required information size to detect or reject the 20% relative risk reduction found in random-effects model meta-analysis is calculated using diversity found in meta-analysis, with double-sided α = 0.05 and β = 0.20 (power of 80%).

TSA was performed for all the outcomes without significant heterogeneity. The Z value is the test statistic and |Z| = 1.96 corresponds to a *P* = .05, with greater Z values corresponding to lower *P* values. The Z-score curve for mortality (adjusted RR 1.0 [0.77-1.31], *P* = .98; I^2^ = 0%; Figure 4, *A*), MI (adjusted RR 0.91 [0.74-1.11], *P* = .35; I^2^ = 0%; Figure 4, *B*), LCOS (adjusted RR, 1.19 [0.59-2.40], *P* = .61; I^2^ = 46%; Figure 4, *C*), and AF (adjusted RR, 1.07 [0.86-1.33], *P* = .49; I^2^ = 28%; Figure 4, *D*) crossed the required information size boundary, indicating current evidence was sufficient in concluding that there were no significant differences between both arms. The Z-score curve for IABP use (adjusted RR, 0.99 [0.60-1.64], *P* = .96; I^2^ = 20%; Figure 5, *A*), stroke (adjusted RR, 1.03 [0.57-1.87], *P* = .89; I^2^ = 0%; Figure 5, *B*), and AKI (adjusted RR, 0.97 [0.51-1.84], *P* = .92; I^2^ = 23%; Figure 5, *C*) crossed the futility boundary, indicating current evidence was sufficient in ruling out a 20% RR reduction in these outcomes.

## Discussion

**Figure 6:**
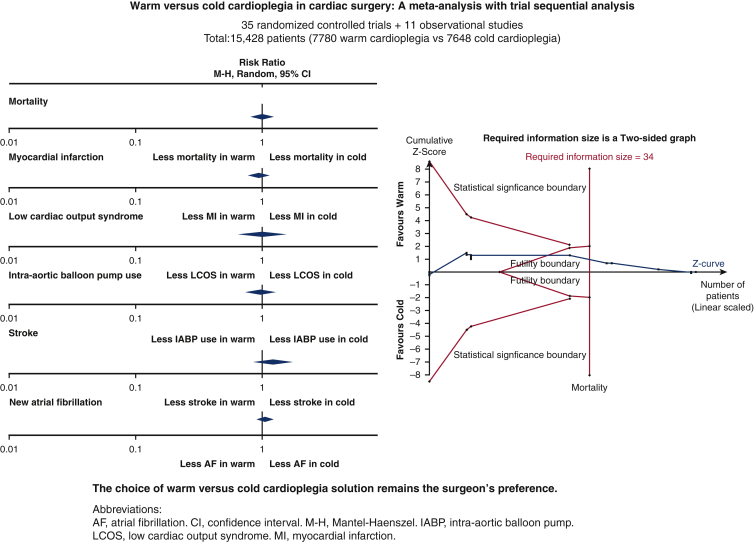
Warm versus cold cardioplegia in cardiac surgery: a meta-analysis with trial sequential analysis. Forty-six studies, with 15,428 patients were included in analysis (35 randomized controlled trials + 11 observational studies). No significant differences were found between two arms in post-operative mortality, myocardial infarction, low cardiac output syndrome, intra-aortic balloon pump use, stroke, and new atrial fibrillation as shown in the Forest plots. Trial sequential analysis of mortality was shown signifying current evidences were conclusive. In conclusion, choice of warm versus cold cardioplegia remains surgeon's preference.

In this study, we compared operative and clinical outcomes of warm and cold cardioplegia. No significant differences were found between both arms for all outcomes. TSA showed that current evidence was conclusive to rule out 20% RR reduction in the following outcomes: mortality, MI, LCOS, IABP use, stroke, new AF, and AKI (Figure 6).

Overall, our results confirmed the findings by Fan and colleagues^3^ that warm and cold cardioplegia were not significantly different in efficacy and safety, further providing a broader look at clinical and operative outcomes. Despite not exhibiting statistically significant subgroup differences, diverging trends were found upon subgroup analysis, suggestive of subtle differences between the subgroups. However, when analyzing only studies with low risk of bias, these numerical trends disappeared. This suggests that such trends might have been the result of bias, possibly due to unclear or inappropriate randomization methods and a lack of blinding in some trials. Furthermore, when we compared cold blood with cold crystalloid cardioplegia, the outcomes of mortality, LCOS, IABP use, stroke, and AKI showed trends in opposite directions, with subgroup differences of *P* = .27, *P* = .21, *P* = .009, *P* = .09, and *P* = .08, respectively. The 2014 meta-analysis by Zeng and colleagues^10^ suggested that subtle subgroup differences can lead to drastically different outcomes. In their study, there were significantly less postoperative MI in cold blood cardioplegia; however, there were no significant differences in mortality, AF, and stroke between cold blood versus crystalloid cardioplegia. This may have been the reason for the statistically insignificant trends in our subgroup analysis, as current studies may not have been designed to specifically compare cold blood versus cold crystalloid cardioplegia. Nonetheless, the results by Zeng and colleagues^10^ were limited by high risks of bias and other possible confounders; therefore, more studies are needed to evaluate the effects of blood versus crystalloid cardioplegia.

All primary outcomes and AKI exhibited insignificant heterogeneity, whereas TSA showed conclusive results. While all primary outcomes were supported by at least 15 studies, suggesting uniformity of the included studies regarding the outcome of interest. In contrast, hospital LOS and ICU LOS displayed significant heterogeneity. This could be contributed by several factors, including differences in local practices, the admission and discharge criteria of ICU, experience of surgeons' etcetera. Heterogeneity remained high despite stratification by subgroups, suggesting that variability was less likely to be caused by differences in publication year, biased studies, type of surgery, or composition of cardioplegia solution. Although such significant heterogeneity limited the strength of our findings, our analysis represented the most up-to-date evidence. However, there is a need for further studies delineating factors affecting the aforementioned outcomes, along with trials controlling for the aforementioned factors.

A survey performed by Ali and colleagues^11^ in 2018 revealed significant variation in the international practice of myocardial protection, with no clear consensus on the use of cardioplegia currently. Variability exists in composition and delivery method of cardioplegic solutions. However, limited by evidences available, subgroup analysis was not performed in those aspects mentioned. Most of our included studies administered cardioplegic solutions in antegrade fashion. Composition of blood and crystalloid solutions varies among studies, including mixture of blood with other solutions, Custodiol solution, Buckberg solution, St Thomas Hospital solution, and Del Nido solution, etc. Further studies should be done focusing on factors that were not evaluated in this meta-analysis.

The safety and efficacy of different types of crystalloid solutions (eg, histidine–tryptophan–ketoglutarate solution, St Thomas solution) warrants further investigations, as exemplified in the study by Pizano and colleagues.^12^ Histidine-tryptophan-ketoglutarate solution is a widely used cardioplegic and organ-preserving solution; however, despite its widespread use, it is seldom studied in comparison with blood cardioplegia. Del Nido solution was initially intended for pediatric surgeries and is now extended to adult cardiac surgeries. Ler and colleagues,^13^ in a meta-analysis performed in 2020, compared Del Nido versus St Thomas cardioplegic solution, showing similar postoperative outcomes.

Besides composition of cardioplegic solution, mode of administration should be further explored. Gambardella and colleagues^14^ performed a meta-analysis in 2019 comparing single versus multidose cardioplegia, suggesting that more studies were needed to compare effects of different solution types, as current evidence were not yet conclusive. In addition, terminal hot-shot cardioplegia was proposed as a potential way to improve clinical outcomes, yet a systematic review performed by Volpi and colleagues^15^ in 2019 concluded that there was insufficient evidence to evaluate its clinical merits. Mallidi and colleagues^16^ conducted an observational study, suggesting that warm blood cardioplegia was associated with better long-term survival and less late MI than cold blood cardioplegia. However, there are few studies in the current literature that investigate the long-term effects of warm versus cold cardioplegia, which in fact are important concerns that should be further explored.

In fact, the choice of cardioplegic solutions might be based on other perceived benefits that were not investigated in this meta-analysis. At our unit, which consisted of 22 consultant cardiac and aortic surgeons, choice of cardioplegic temperature remained surgeons’ preferences. The majority of our team members preferred cold cardioplegia, aiming for metabolic inhibition and thus less energy consumption. In the meta-analysis by Ler and colleagues,^13^ the rate of defibrillation, aortic crossclamp time, and cardiopulmonary bypass time were listed as primary outcomes. This might suggest future directions of investigating for other outcomes.

This meta-analysis has a few limitations. First, one major confounder was the variation in means of administrating the cardioplegia solutions: intermittent versus continuous and antegrade versus retrograde. This meta-analysis was not designed to investigate these factors; thus, it was unclear the impact of these factors on our findings. Second, a significant proportion of the studies included that were published before 2009 had a high risk of bias; however, we attempted to mitigate this issue by performing a subgroup analysis of studies with low risk of bias. Third, the results of observational studies published after 2009 were pooled with RCTs, which may add on to confounders and selection bias. To address this issue, we performed a subgroup analysis for RCTs, which showed no changes of statistical significances of all outcomes. In addition to that, the observational studies included in this meta-analysis were of low risk of bias, and their value lies in their representation of real-world experience. Fourth, this study based on and updated the findings of previously meta-analysis by Fan and colleagues in 2010.^3^ The results might therefore be inaccurate if it was of poor quality. Thus, a quality assessment was performed, showing that it was of moderate quality without major methodologic flaws. Fifth, the studies included in this meta-analysis spanned over 27 years, during which time surgical techniques and clinical practices have advanced significantly. To mitigate this issue, we performed a subgroup analysis of studies published after 2009. Nonetheless, this cutoff of publication time was merely based on the publication of the last meta-analysis on this topic, and the time of publication remains a potential confounder. Finally, only studies in English language were included; therefore, it is possible that relevant non-English studies were omitted.

In conclusion, this systematic review and meta-analysis concluded that there were no significant differences in postoperative rates of mortality, MI, LCOS, IABP use, stroke, new AF, and AKI, between the use of warm and cold cardioplegia. The choice of warm versus cold cardioplegia solution remains the surgeon's preference. Nonetheless, further studies should evaluate any differences between various compositions and modes of administrating cardioplegic solutions, with greater exploration on the long-term effects of warm versus cold cardioplegia.

### Conflict of Interest Statement

The authors reported no conflicts of interest. The funding was provided by University College London Open Access Team, Main Library; University College London, UK.

The *Journal* policy requires editors and reviewers to disclose conflicts of interest and to decline handling or reviewing manuscripts for which they may have a conflict of interest. The editors and reviewers of this article have no conflicts of interest.

## Figures and Tables

**Table E1:** Critical appraisal of the included randomized controlled trials using the modified Jadad scale

Authors	Was the study described as randomized? Yes: 1 mark No: 0 mark	Was the method of randomization appropriate? Yes: 1 mark No: –1 mark Not described: 0 mark	Was the study described as blinded? Double-blind: 1 mark Single-blind: 0.5 mark No: 0 mark	Was the method of blinding appropriate? Yes: 1 mark No: –1 mark Not described: 0 mark	Was there a description of withdrawals and dropouts? Yes: 1 mark No: 0 mark	Was there a clear description of the inclusion/exclusion criteria? Yes: 1 mark No: 0 mark	Was the method used to assess adverse effects described? Yes: 1 mark No: 0 mark	Were the methods of statistical analysis described? Yes: 1 mark No: 0 mark	Total score (out of 8)
Ali et al 1994^E2^	1	–1	0	0	0	1	1	1	3
Ascione et al 2002^E3^	1	0	0	0	1	1	1	1	5
Baron et al 2003^E4^	1	0	0	0	0	0	1	1	3
Chello et al 1997^E5^	1	–1	0	0	0	0	1	1	2
Chello et al 2003^E6^	1	0	0	0	1	0	1	1	4
Chocron et al 2000^E7^	1	1	0	0	1	1	1	1	6
Curtis et al 1996^E8^	1	0	0	0	1	0	1	1	4
Dar et al 2005^E9^	1	0	0	0	0	1	1	1	4
Elwatidy et al 1999^E10^	1	0	0	0	1	0	1	1	4
Engelman et al 1996^E11^	1	1	1	1	1	1	1	1	8
Franke et al 2003^E12^	1	1	0	0	1	1	1	1	6
Gaudino et al 2013^E13^	1	1	1	0	1	1	1	1	7
Hayashida et al 1994^E14^	1	1	0	0	1	0	0	1	4
Hayashida et al 1995^E15^	1	1	0	0	0	0	1	1	4
Honkonen et al 1997^E16^	1	0	0	0	0	1	1	1	4
Isomura et al 1995^E17^	1	0	0	0	0	0	1	1	3
Jacquet et al 1999^E18^	1	0	0	0	1	1	1	1	5
Kammerer et al 2010^E19^	1	0	0	0	0	1	1	1	4
Kuhn et al 2015^E20^	1	1	1	0	1	1	1	1	7
Lajos et al 1993^E21^	1	–1	0	0	1	0	1	1	3
Landymore et al 1996^E22^	1	1	0	0	0	1	1	1	5
Maccherini et al 1995^E23^	1	0	0	0	0	0	1	0	2
Martin et al 1994^E24^	1	1	0	0	1	0	0	1	4
Pelletier et al 1994^E25^	1	1	0.5	1	0	1	1	1	6.5
Pepper et al 1995^E26^	1	0	0.5	0	0	0	1	1	3.5
Rashid et al 1994^E27^	1	–1	0	0	0	0	1	1	2
Rashid et al 1995^E28^	1	–1	0	0	0	0	1	1	2
Saclı et al 2019^E29^	1	–1	0	0	0	1	1	1	3
Şirlak et al 2003^E30^	1	0	0	0	1	1	1	1	5
Sirvinskas et al 2005^E31^	1	1	0	0	1	1	1	1	6
The Warm Heart Investigator 1994^E32^	1	1	0.5	0	1	1	1	1	6.5
Ucak et al 2019^E33^	1	1	0	0	0	1	1	1	5
Yau et al 1992^E34^	1	1	0	0	0	0	0	1	3
Yau et al 1993^E35^	1	1	0	0	1	0	1	1	5
Yang et al 1994^E36^	1	0	0	0	0	0	0	1	2

**Table E2:** Critical appraisal of the included observational studies using the Newcastle–Ottawa Quality Assessment Scale

Authors	Selection				Comparability	Outcome			Total score (out of 9)
	Does the study clearly show that the people with the outcome were excluded from the study sample? Yes: 1 mark No: 0 mark	How representativeness is the exposed group of the typical person in the community? Truly/somewhat representative of the average in the community: 1 mark Select, unrepresentative group, not described: –0 mark	Selection of the non-exposed cohort Same community as exposed cohort: 1 mark Different source, no description: 0 mark	Was exposure status ascertained in a way that prevents/minimizes misclassification of exposure status? Secure or unbiased record/structured interview: 1 mark Written self-report, interview without validated questionnaire, no description: 0 mark	Does the study adequately deal with potential confounders? Study controls for most important confounding factor: 1 mark Study further controls for any additional potential confounding factor: 1 mark	Assessment of outcome Independent or blind assessment stated in the paper, or confirmation of the outcome by reference to secure records/record linkage (ICD): 1 mark Self-report, no descriptions: 0 mark	Based on your clinical judgement, was FU long enough for outcomes to occur? Yes: 1 mark No: 0 mark	Adequacy of FU of cohorts Complete FU/subjects lost to FU unlikely to introduce bias: 1 mark No description: 0 mark	
Raza Baig et al 2015^E37^	1	1	1	1	2	1	1	1	9
Candilio et al 2014^E38^	1	1	1	1	2	1	1	1	9
De Jonge et al 2015^E39^	0	1	1	1	2	1	1	1	8
Kuhn et al 2018^E40^	0	1	1	1	2	1	1	1	8
Mourad et al 2016^E41^	1	1	1	1	2	1	1	1	9
Nardi et al 2018^E42^	0	1	1	1	2	1	1	1	8
Nardi et al 2018^E43^	0	1	1	1	2	1	1	1	8
Plicner et al 2017^E44^	1	1	1	1	2	1	1	1	9
Rosu et al 2012^E45^	0	1	1	1	2	1	1	1	8
Trescher et al 2017^E46^	0	1	1	1	2	1	1	1	8
Zeriouh et al 2015^E47^	1	1	1	1	2	1	1	1	9

*ICD*, *International Classification of Diseases*; *FU*, follow-up.

**Table E3:** Baseline characteristics of patients included after previous meta-analysis

	OR or WMD [95% CI]	*P* value	Reporting studies, n (%)
Age	WMD 0.01 [–0.43, 0.45]	.98	13 (81.25%)
Male	OR 0.74 [0.62-0.89]	.001	15 (93.75%)
Hypertension	OR 1.05 [0.86-1.29]	.63	11 (68.75%)
DM	OR 1.08 [0.97-1.19]	.15	11 (68.75%)
LVEF	WMD –0.15 [–1.64, 1.34]	.85	10 (62.50%)

*OR*, Odds ratio; *WMD*, weighted mean difference; *CI*, confidence interval; *DM*, diabetes mellitus; *LVEF*, left ventricular ejection fraction.

**Table E4:** Pairing table

Author	Year	Mortality	MI	LCOS	IABP use	Stroke	New AF	AKI	Hospital LOS	ICU LOS	Risk of bias -MJS (/8) NOS (/9)
Ali et al^E2^	1994		✓	✓							3/8
Ascione et al^E3^	2002		✓				✓				5/8
Raza Baig et al^E37^	2015		✓								9/9
Baron et al^E4^	2003	✓					✓				3/8
Candilio et al^E38^	2014	✓	✓			✓	✓	✓	✓	✓	9/9
Chello et al^E5^	1997			✓							2/8
Chello et al^E6^	2003			✓							4/8
Chocron et al^E7^	2000		✓			✓					6/8
Curtis et al^E8^	1996	✓	✓								4/8
De Jonge et al^E39^	2015	✓			✓					✓	8/9
Elwatidy et al^E10^	1999		✓	✓	✓						4/8
Engelman et al^E11^	1996		✓		✓		✓				8/8
Franke et al^E12^	2003	✓	✓		✓	✓	✓				6/8
Gaudino et al^E13^	2013	✓				✓			✓	✓	7/8
Hayashida et al^E14^	1994		✓	✓	✓	✓					4/8
Hayashida et al^E15^	1995		✓								4/8
Honkonen et al^E16^	1997		✓								4/8
Isomura et al^E17^	1995		✓								3/8
Jacquet et al^E18^	1999	✓	✓		✓	✓					5/8
Kammerer et al^E19^	2010	✓				✓					4/8
Kuhn et al^E20^	2015	✓					✓			✓	7/8
Kuhn et al^E40^	2018	✓				✓	✓		✓		8/9
Lajos et al^E21^	1993	✓	✓		✓	✓	✓				3/8
Landymore et al^E22^	1996	✓	✓		✓						5/8
Maccherini et al^E23^	1995	✓									2/8
Martin et al^E24^	1994	✓	✓		✓	✓					4/8
Mourad et al^E41^	2016		✓							✓	9/9
Nardi et al^E42^	2018	✓	✓	✓	✓	✓	✓	✓	✓	✓	8/9
Nardi et al^E43^	2018	✓	✓	✓	✓		✓	✓	✓	✓	8/9
Pelletier et al^E25^	1994	✓	✓			✓					6.5/8
Pepper et al^E26^	1995	✓									3.5/8
Plicner et al^E44^	2017	✓	✓								9/9
Rashid et al^E27^	1994	✓	✓		✓		✓				2/8
Rashid et al^E28^	1995	✓	✓		✓		✓				2/8
Rosu et al^E45^	2012	✓	✓	✓	✓	✓	✓	✓	✓	✓	8/9
Saclı et al^E29^	2019	✓		✓			✓	✓	✓	✓	3/8
Şirlak et al^E30^	2003			✓	✓						5/8
Sirvinskas et al^E31^	2005	✓	✓		✓		✓				6/8
The Warm Heart Investigators^E32^	1994	✓	✓			✓	✓	✓			6.5/8
Trescher et al^E46^	2017	✓	✓	✓	✓	✓	✓	✓	✓	✓	8/9
Ucak et al^E33^	2019	✓	✓	✓	✓	✓					5/8
Yau et al^E34^	1992	✓	✓	✓							3/8
Yau et al^E35^	1993	✓	✓	✓	✓	✓					5/8
Yang et al^E36^	1994	✓		✓							2/8
Zeriouh et al^E47^	2015	✓	✓		✓				✓	✓	9/9

*MI*, Myocardial infarction; *LCOS*, low cardiac output syndrome; *IABP*, intra-aortic balloon pump; *AF*, atrial fibrillation; *AKI*, acute kidney injury; *ICU*, intensive care unit; *LOS*, length of stay; *MJS*, modified Jadad scale; *NOS*, Newcastle–Ottawa Quality Assessment Scale.

**Table E5:** Summary of primary outcomes since publication of previous meta-analysis

	RR [95% CI]	*P* value	Subgroup differences (*P* value)
Mortality	1.09 [0.85-1.41]	.50	.16
MI	1.05 [0.71-1.57]	.80	.54
LCOS	1.60 [0.72-3.55]	.25	.16
IABP use	0.71 [0.35-1.42]	.33	.21
Stroke	0.98 [0.59-1.64]	.94	.32
New AF	1.10 [0.87-1.39]	.41	.87

*RR*, Relative risk; *CI*, confidence interval; *MI*, myocardial infarction; *LCOS*, low cardiac output syndrome; *IABP*, intra-aortic balloon pump; *AF*, atrial fibrillation.

**Table E6:** Summary of primary and secondary outcomes from low risk of bias studies

	RR or WMD [95% CI]	*P* value	Subgroup differences (*P* value)
Mortality	RR 1.00 [0.79-1.27]	.99	.85
MI	RR 0.92 [0.76-1.12]	.42	.67
LCOS	RR 1.19 [0.61-2.31]	.61	.45
IABP use	RR 0.95 [0.65-1.39]	.80	.89
Stroke	RR 0.98 [0.67-1.44]	.92	.01
New AF	RR 1.07 [0.89-1.29]	.49	.75
AKI	RR 0.97 [0.57-1.66]	.92	.70
Hospital LOS	WMD –0.84 [–1.59, –0.10]	.03	.07
ICU LOS	WMD –0.18 [–0.63, 0.27]	.43	.15

*RR*, Relative risk; *WMD*, weighted mean difference; *CI*, confidence interval; *MI*, myocardial infarction; *LCOS*, low cardiac output syndrome; *IABP*, intra-aortic balloon pump; *AF*, atrial fibrillation; *AKI*, acute kidney injury; *LOS*, length of stay; *ICU*, intensive care unit.

**Table E7:** Summary of primary and secondary outcomes from studies with coronary artery bypass graft performed

	RR or WMD [95% CI]	*P* value	Subgroup differences (*P* value)
Mortality	RR 0.95 [0.74-1.21]	.65	.66
MI	RR 0.88 [0.73-1.07]	.21	.08
LCOS	RR 1.00 [0.62-1.61]	1.00	.12
IABP use	RR 0.95 [0.70-1.28]	.72	N/A
Stroke	RR 1.22 [0.80-1.87]	.35	.77
New AF	RR 1.05 [0.86-1.28]	.64	.50
AKI	RR 0.84 [0.40, 1.76]	.65	.51
Hospital LOS	WMD –0.69 [–1.66, 0.28]	.16	.18
ICU LOS	WMD –0.04 [–0.56, 0.48]	.89	.43

*RR*, Relative risk; *WMD*, weighted mean difference; *CI*, confidence interval; *MI*, myocardial infarction; *LCOS*, low cardiac output syndrome; *IABP*, intra-aortic balloon pump; *N/A*, not available; *AF*, atrial fibrillation; *AKI*, acute kidney injury; *LOS*, length of stay; *ICU*, intensive care unit.

**Table E8:** Summary of primary and secondary outcomes from studies with CB and CC cardioplegia

	RR or WMD [95% CI], *P* value	Subgroup differences
Mortality	CB: RR 1.09 [0.81-1.46], *P* = .57 CC: RR 0.85 [0.61-1.19], *P* = .34	I^2^ = 17.4%, χ^2^ = 1.21, *P* = .27
MI	CB: RR 0.91 [0.74-1.11], *P* = .33 CC: RR 1.16 [0.68-1.98], *P* = .58	I^2^ = 0%, χ^2^ = 0.75, *P* = .39
LCOS	CB: RR 1.25 [0.66-2.34], *P* = .49 CC: RR 0.68 [0.33-1.38], *P* = .28	I^2^ = 36.9%, χ^2^ = 1.58, *P* = .21
IABP use	CB: RR 1.49 [0.94-2.36], *P* = .09 CC: RR 0.65 [0.43-0.99], *P* = .04	I^2^ = 85.2%, χ^2^ = 6.78, *P* = .009
Stroke	CB: RR 0.96 [0.64-1.45], *P* = .86 CC: RR 1.95 [0.97-3.93], *P* = .06	I^2^ = 65.5%, χ^2^ = 2.90, *P* = .09
New AF	CB: RR 1.02 [0.87-1.21], *P* = .78 CC: RR 1.16 [0.79-1.71], *P* = .45	I^2^ = 0%, χ^2^ = 0.34, *P* = .56
AKI	CB: RR 0.74 [0.43-1.26], *P* = .26 CC: RR 1.92 [0.76-4.87], *P* = .17	I^2^ = 67.5%, χ^2^ = 3.08, *P* = .08
Hospital LOS	CB: WMD –0.62 [–1.89, 0.64], *P* = .33 CC: WMD –0.58 [–1.44, 0.27], *P* = .18	I^2^ = 0%, χ^2^ = 0.00, *P* = .96
ICU LOS	CB: WMD –0.06 [–1.33, 1.21], *P* = .93 CC: WMD –0.16 [–0.46, 0.14], *P* = .30	I^2^ = 0%, χ^2^ = 0.02, *P* = .88

*CB*, Cold blood; *CC*, cold crystalloid; *RR*, relative risk; *WMD*, weighted mean difference; *CI*, confidence interval; *MI*, myocardial infarction; *LCOS*, low cardiac output syndrome; *IABP*, intra-aortic balloon pump; *AF*, atrial fibrillation; *AKI*, acute kidney injury; *LOS*, length of stay; *ICU*, intensive care unit.

## Supplementary Data

PRISMA 2009 checklist.doc

AMSTAR - Assessing the Methodological Quality of Systematic Reviews
